# Cytokines and Hepatocellular Carcinoma: Biomarkers of a Deadly Embrace

**DOI:** 10.3390/jpm13010005

**Published:** 2022-12-20

**Authors:** Krizia Pocino, Annunziata Stefanile, Valerio Basile, Cecilia Napodano, Francesca D’Ambrosio, Riccardo Di Santo, Cinzia Anna Maria Callà, Francesca Gulli, Raffaele Saporito, Gabriele Ciasca, Francesco Equitani, Umberto Basile, Mariapaola Marino

**Affiliations:** 1 Unità Operativa Complessa Patologia Clinica, Ospedale Generale di Zona San Pietro Fatebenefratelli, 00189 Rome, Italy; 2 Clinical Pathology Unit and Cancer Biobank, Department of Research and Advanced Technologies, IRCCS Regina Elena National Cancer Institute, 00144 Rome, Italy; 3 Synlab Data Medica, 35133 Padova, Italy; 4 Dipartimento di Scienze di Laboratorio e Infettivologiche, Università Cattolica del Sacro Cuore, 00168 Rome, Italy; 5 Fondazione Policlinico Universitario “A. Gemelli” IRCCS, 00168 Rome, Italy; 6 Dipartimento di Neuroscienze, Sezione di Fisica, Università Cattolica del Sacro Cuore, Fondazione Policlinico Universitario “A. Gemelli” IRCCS, 00168 Rome, Italy; 7 Clinical Biochemistry Laboratory, IRCCS “Bambino Gesù” Children’s Hospital, 00165 Rome, Italy; 8 Dipartimento di Medicina Trasfusionale, Ospedale Santa Maria Goretti, AUSL Latina, 04100 Latina, Italy; 9 Dipartimento di Patologia Clinica, Ospedale Santa Maria Goretti, AUSL Latina, 04100 Latina, Italy; 10 Dipartimento di Medicina e Chirurgia Traslazionale, Sezione di Patologia Generale, Università Cattolica del Sacro Cuore, 00168 Rome, Italy

**Keywords:** biomarkers, cytokines, hepatocellular carcinoma, personalized medicine

## Abstract

Hepatocellular carcinoma (HCC) represents a worldwide health matter with a major care burden, high prevalence, and poor prognosis. Its pathogenesis mainly varies depending on the underlying etiological factors, although it develops from liver cirrhosis in the majority of cases. This review summarizes the role of the most interesting soluble factors as biomarkers for early diagnosis and as recommended targets for treatment in accordance with the new challenges in precision medicine. In the premalignant environment, inflammatory cells release a wide range of cytokines, chemokines, growth factors, prostaglandins, and proangiogenic factors, making the liver environment more suitable for hepatocyte tumor progression that starts from acquired genetic mutations. A complex interaction of pro-inflammatory (IL-6, TNF-α) and anti-inflammatory cytokines (TGF-α and -β), pro-angiogenic molecules (including the Angiopoietins, HGF, PECAM-1, HIF-1α, VEGF), different transcription factors (NF-kB, STAT-3), and their signaling pathways are involved in the development of HCC. Since cytokines are expressed and released during the different stages of HCC progression, their measurement, by different available methods, can provide in-depth information on the identification and management of HCC.

## 1. Introduction

Hepatocellular carcinoma (HCC) is the most frequent type of cancer affecting the liver, and its incidence almost exceeds mortality. All those risk factors (chronic HBV or HCV infection, alcohol, aflatoxin B1, NAFLD/NASH) that concur with liver cirrhosis may be involved in HCC pathogenesis [1].

Cirrhotic liver tissue is characterized by low levels of hepatocyte cell proliferation in favor of a greater abundance of inflammatory mediators, fibrosis, and activation of the extracellular matrix environment. Therefore, a hepatocyte clone with a deregulated proliferative rate finds more suitable conditions for expansion, unlike in a normal and proliferating liver [2].

Following a viral infection or toxic tissue damage, a tightly regulated and coordinated multistep process may start in the liver, characterized by activation and local infiltration of immune cells and subsequent engagement in tissue repair. In this refined orchestration of events, the release of a wide range of soluble factors takes place [3]. 

**Figure 1:**
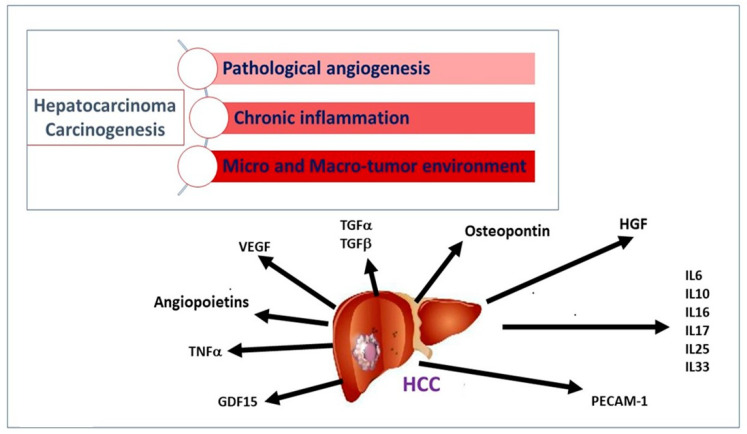
Major pathogenetic events and cytokine network involved in hepatocellular carcinogenesis. Following persistent liver damage, locally activated chronic inflammation favors the release of soluble factors sustaining the proliferation and survival of tumor cells. Angiogenic factors (angiopoietins, VEGF), growth factors for normal and transformed hepatocytes (HGF), adhesion molecules and cytokines for recruitment and activation of leukocytes (PECAM, IL-6), and stellate cells upregulates TGF in the liver and, consequently, regeneration, proliferation, and hepatocyte dysplasia, and, ultimately, the development of HCC.

In liver cirrhosis, a wide proliferation of stellate cells has been described, generating an abundance of extracellular matrix proteins, cytokines, growth factors, and oxidative stress products. The unbalanced expression of these factors and the initial unresolved inflammatory response produce a suitable microenvironment for developing neoplasm. Cytokines released by the tumor, neighboring non-tumor cells, and immune cells can act as a promoter of tumor survival [4] (Figure 1). Carcinogenetic events of HCC involve angiogenesis, chronic inflammation, and tumor micro and macro-environment (Figure 1).

Since cytokines production regulates HCC evolution and worsening progression, their evaluation can provide useful information on the identification and management of HCC.

## 2. Cytokines and Growth Factors

### 2.1. Stimulators of Angiogenesis and Tumor Invasiveness

The progression of liver disease takes into account pathological angiogenesis, a prerequisite that facilitates the development of HCC. Angiogenesis is the result of a multiphase process and is the limiting step of tumor growth. In normal conditions, there is a balance between angiogenic inducers and inhibitors that keeps the angiogenic process under control and prevents inappropriate tissue vascularization. Angiogenesis inhibitors often derive from circulating extracellular matrix proteins (because of injury to the matrix), e.g., fibronectin, prolactin, collagen XVIII (endostatin), Hepatocyte Growth Factor fragment NK1, and angiostatin. Although tumors initially engage the pre-existing vascularity, an angiogenetic “switch” consisting of the production of factors inducing angiogenesis crucially modifies the tumor phenotype [5].

**Figure 2:**
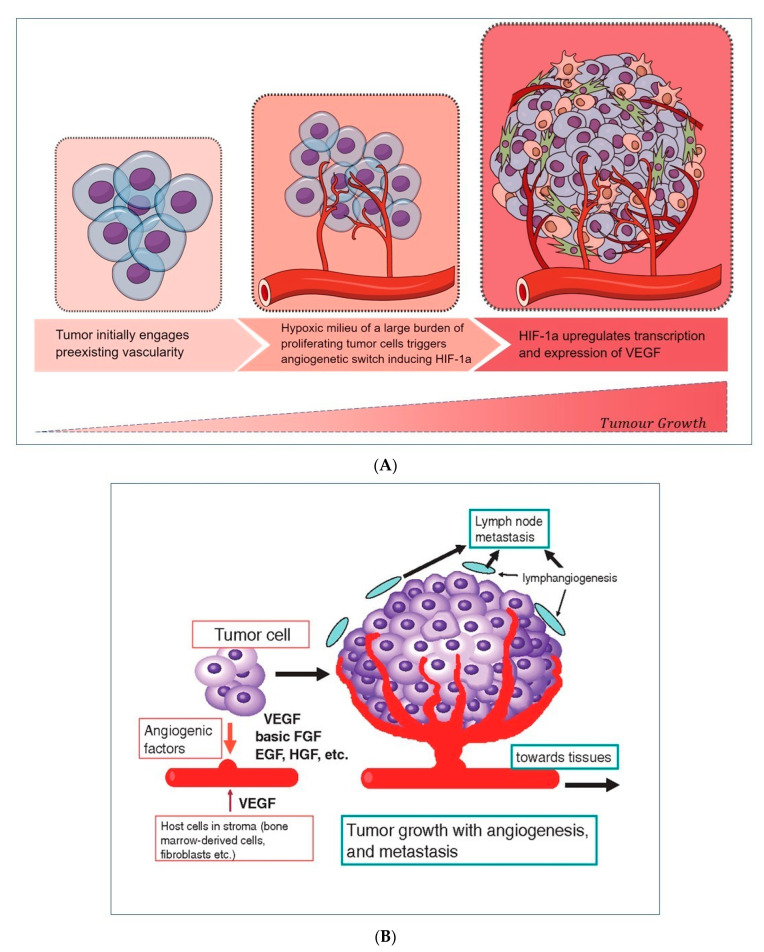
(**A**) Hypoxic environment stimulates neoangiogenesis, thanks to a circuit involving HIF-1α -induced VEGF release by tumor cells. (**B**) A wide range of cellular released growth factors, derived from a tumor, stroma, and leukocytes, ensures the formation of new vascularity and the sustainment of cell growth, allowing the worsening progression of the initial tumor burden.

Vascular endothelial growth factor (VEGF) is the most powerful stimulator of normal and pathological angiogenesis (Table 1). Circulating VEGF may be derived mainly from the large burden of tumor cells released under hypoxic conditions. Its expression is regulated by the hypoxia-inducible factor 1α (HIF-1α), which, induced during the hypoxic conditions, triggers the transcription of VEGF that stimulates the formation of new vessels (Figure 2A) [6]. This indicates that VEGF participates in the initial phase of angiogenesis. As a result, the transition of endothelial cells from an inactive to an active state can occur along with their proliferation, migration, and formation of new vessels, which can act as new gates for the recruitment of inflammatory cells, releasing cytokines and inducing further inflammation (Figure 2B). Different reports analyzed serum levels of VEGF in HCC patients in comparison to patients with or without HCV-related cirrhosis, often with opposite results [7,8]. Mukozu et al. showed that VEGF was higher in HCC patients compared to controls [7], while the results from Abden–Ramahal et al. displayed significantly higher serum levels of VEGF in HCC in comparison to cirrhotic patients, but no significant differences in healthy controls [8]. However, altogether these data highlight the important role of VEGF as a biomarker of vascular invasion in disease progression from liver cirrhosis to HCC.

Patients with HCC showed a significant increase in VEGF after anticancer therapy compared to the values reported at the time of diagnosis, as well as to the levels of lymphocytes [9]. This may be partly explained by the rebound effect of VEGF, induced by hypoxia following locoregional treatments, often associated with treatment failure and low survival rates in patients [10]. Levels of VEGF are increased in patients who later experienced progression of HCC compared to those who remained stable [11]. Additionally, higher VEGF levels prior to sorafenib treatment (a multikinase inhibitor employed in several locally recurrent or metastatic solid tumors, including HCC) are associated with shorter survival [12,13]. 

**Table 1:** Role of the main cytokines and growth factors in hepatocarcinogenesis.

Biomarkers	Abbreviation	Role in HCC	Study
Vascular endothelial growth factor	VEGF	◊ Regulates initial phase of angiogenesis. ◊ Higher VEGF levels associated with poor outcomes in HCC patients (prior to sorafenib treatment). ◊ Indicator of clinical efficacy	Tammela, T. et.al., 2005 [6]
Angiopoietin/Tie system		◊ Key role during the late phase of angiogenesis, responsible for development of newly established vascular structures. ◊ Their activity determines the stabilization of new vessels.	Naldini, A. et al., 2005 [14]
Tyrosine kinase with Ig and EGF-homology domains-1 and 2	Tie1, Tie2		
Angiopoietin ligands 1–4	Ang1, Ang2, Ang3, Ang4		
Hepatocyte growth factor	HGF	◊ Stimulates the invasiveness of tumor cells. ◊ Correlates with patient survival time and tumor size.	García-Vilas JA, et al., 2018 [15]
Platelet endothelial cell adhesion molecule-1	PECAM-1	◊ Crucial for the angiogenesis process. ◊ Positively correlate with MELD and its identification can help to assess the degree of tumor angiogenesis. ◊ Promotes the formation of metastases.	DeLisser, H.M. et al., 1997 [16]
Interleukin 6	IL-6	◊ Serum levels increased in advanced HCC. ◊ Serum levels associated with lower overall survival and prone to early relapses in HCC patients undergoing hepatectomy. ◊ Elevated concentrations correlate with poor overall survival	He, G. et al., 2013 [17]
Transforming growth factor alpha	TGF-α	◊ Related to the hepatocarcinogenesis. ◊ Correlated with regeneration, proliferation, hepatocyte dysplasia and the development of HCC.	Shao, Y. et al., 2017 [18]
Transforming growth factor beta	TGF-β	◊ Related to the hepatocarcinogenesis. ◊ Regulates many inflammatory processes.	Shao, Y. et al., 2017 [18]
Interleukin 10	IL-10	◊ Powerful anti-inflammatory cytokine. ◊ Serum levels as negative prognostic factor.	Shakiba, E.et al., 2018 [19]
Interleukin 16–33, -17, -25	IL 16-IL-33, IL-17, IL-25	◊ Biomarkers of disease progression.	Cruikshank, W. et al., 2000 [20] Askoura M. et al., 2022 [21]
Growth differentiation factor 15	GDF15	◊ Induced by HCV infection and regulates hepatocellular carcinoma-related genes. ◊ Genetic ablation of GDF-15 has no apparent effect on HCC tumor formation rate, growth rate or invasiveness.	Myojin, Y. et al., 2022 [22]
Tumor necrosis factor	TNF	◊ In liver induces biological responses (apoptosis, necrosis, inflammation, regeneration) and progression of HCC.	Tiegs, G.et al., 2022 [23]
Osteopontin		◊ Increased serum levels are found in individuals with HCC compared to liver cirrhosis alone or chronic liver disease ◊ Diagnostic efficacy in detecting early-stage HCC.	Zhao, H. et al. 2018, [24]

The Tyrosine kinase proteins with Ig and EGF-homology domains 1 and 2 (Tie1 and Tie2) and their angiopoietin ligands 1–4 (Ang1, 2, 3, and 4) play a key role during the late phase of angiogenesis and are responsible for the maturation of newly established vascular structures (Table 1). Ang1 and Ang2 have been deeply studied and characterized. The activity of the Angiopoietin/Tie system determines the stabilization of new vessels [25]. There is growing evidence that the angiopoietin/Tie signal can modify ongoing inflammation [14]. Ang1 appears to be a powerful activator of Tie2, as well as a regulator of blood vessel formation and development. Experimental studies showed that Ang1 acts as an anti-inflammatory molecule [26] but can induce pulmonary hypertension as a complication [27]. Ang1 also neutralizes tissue factor activity that is relevant for the induction of coagulation, thrombosis, and inflammatory response. Furthermore, Ang1 reduces the adhesion of VEGF-related leukocytes to the endothelium [28,29]. Conversely, Ang2 acts as a competitive antagonist of Ang1, deregulates the signal pathway of Tie2 [25], and plays a pro-inflammatory role [30,31]. Additionally, significantly high Ang2 serum levels have been observed in patients during liver carcinogenesis [32]. An increase in Ang2 levels has been observed in correlation with the liver disease progression [9,33], while Ang1 negatively correlates with the model for end-stage liver disease (MELD) and the hepatic fibrosis index. All these data confirm the potential diagnostic utility of Ang1 and Ang2 levels as developing new quantitative biomarkers for staging cirrhosis. Serum Ang2 concentrations decrease significantly after treatment with Direct Antiviral Drugs (DAAs), and, consequently, the Ang2/Ang1 ratio also drops. Ang2 is potentially useful in monitoring antiviral therapy [9]. In a recent study, we reported that patients who died from HCC had significantly lower Ang1 levels than those who did not die, placing Ang1 as a potential prognostic index [9]. 

It has been shown that the Hepatocyte growth factor (HGF) is over-expressed in HCC compared to the normal and cirrhotic liver without signs of neoplasia (Table 1) [9,15,34]. Expression of HGF and its receptor supports the existence of both autocrine and paracrine mechanisms of HGF action in HCC if compared to the unique paracrine mechanism found in normal liver tissue (in the absence of cancer), suggesting that it also plays a role in tumor development and progression [15,34,35]. Stellate cells and myofibroblasts are induced to secrete HGF from tumor cell products, and HGF, in turn, stimulates tumor cell invasiveness. Recent reports show that higher serum HGF levels negatively correlate with patient survival time [36] and positively with tumor size [37].

Furthermore, the comparison of cirrhotic patients with and without HCC suggests that HGF levels are potentially useful for monitoring the onset of HCC after a diagnosis of cirrhosis [9]. Interestingly, patients with lower HGF levels prior to treatment display major benefits from sorafenib therapy in terms of overall survival and time to progression [12]. 

Platelet endothelial cell adhesion molecule-1 (PECAM-1), also known as CD31, is normally found on the surface of endothelial cells, platelets, leukocyte subpopulations, and Kupffer cells [38] (Table 1). This molecule is highly expressed within the vascular compartment but largely concentrated at junctions between adjacent cells, and its receptors mediate these interactions that play a crucial role during angiogenesis. In this context, PECAM-1 can mediate both homophilic and heterophilic adhesion [16]. PECAM-1 has been found to positively correlate with MELD, and its identification may aid in assessing the degree of tumor angiogenesis, which may indicate a rapidly growing tumor [39]. Furthermore, PECAM-1 promotes the formation of metastases by inducing the epithelium-mesenchymal transition in HCC by increasing the regulation of β1 integrin through the FAK (focal adhesion kinase) /Akt signaling pathway [40].

### 2.2. Stimulators of Chronic Inflammation, Liver Fibrosis, and Proliferation

Interleukin (IL)-6 acts as an important inducer of the acute phase response and infection defense in the liver [41]. IL-6 binds to the signal-transducing subunit gp130 on target cells either in complex with the membrane-bound or with the soluble IL-6 receptor to activate intracellular signaling. By the latter ‘trans-signaling’ mechanism, IL-6 can target monocyte chemotaxis and maintain sustained chronic inflammation towards any injured tissue [42,43]. Increased serum levels of IL-6 have been found in patients with advanced HCC compared to those with the early stage [44] (Table 1). Additionally, elevated serum IL-6 levels in HCC patients undergoing hepatectomy are associated with lower overall survival and are prone to early relapses [45]. Research results from mouse models of HCC have shown that isolated HCC progenitor cells can give rise to cancer in the presence of ongoing liver damage and that these cells promote their own growth and progress to malignancy via autocrine IL-6 signaling [17]. A clinical study analyzing 128 HCC patients treated with sorafenib evaluated the prognostic value of serum IL-6 levels before treatment. Elevated pretreatment IL-6 concentrations have been found to be an independent predictor of poor overall survival, although there is no association with the efficacy of sorafenib [18].

Transforming growth factors (TGF)-α and TGF-β are closely related to the hepatocarcinogenesis process (Table 1). Normal hepatocytes show low TGF-α expression compared to tumor cells. In fact, following chronic inflammation due to persistent liver damage, the secreted cytokine pool upregulates TGF-α in the liver and, consequently, regeneration, proliferation, hepatocyte dysplasia, and, ultimately, the development of HCC [46]. TGF-β is a key regulator of the late phase of inflammatory processes, not only promoting tissue repair but also inhibiting leukocyte activation and infiltration, acting at least in part using control of adhesion molecules on parenchymal cells [47]. In this way, TGF-β counteracts the effects of proinflammatory cytokines, leading to the inhibition of cellular processes, such as proliferation, differentiation, and survival. Paradoxically, cancer cells may exploit these microenvironment modifications to their advantage [48]. During carcinogenesis, malignant cells can often attenuate the suppressive TGF-β signaling by altering the expression of its receptors but also hijacking the signaling cascade. HCC cell lines with metastatic potential have been described to downregulate TGF-βR2. Interestingly, a reduced expression of TGF-βR2 in HCC correlates with larger tumor size and various metastatic features, such as poor differentiation, portal vein invasion, and intrahepatic metastases [49,50]. In the early stages of cancer, TGF-β acts as a tumor suppressor by inducing cytostasis and apoptosis, while in the later stages, it promotes pro-tumorigenic events, such as the transition of epithelial cells to mesenchymal, invasion, metastasis, and angiogenesis [51]. The simultaneous exposure (or addition) of TGF-β and IL-6 to human HCC cell cultures (Huh) highlighted an attenuation of the pro-proliferative effects induced by IL-6 by TGF-β. This explains a decrease in the transcription levels of the IL-6 receptor (IL-6R), in the expression of STAT-3 (signal transducer and activator of transcription) induced by IL-6, its nuclear localization, and, finally, a reduced activation of p65 compared to the unperturbed activation of the pathway. SMAD (small mother against decapentaplegic)-dependent TGF-β, resulting in the transition from epithelial to mesenchymal cells and, thus, loss of cell polarity and cell adhesion, as well as the acquisition of invasive and migratory properties, coupled with cell growth arrest [52].

IL-10 is a potent anti-inflammatory cytokine (Table 1). Its role in HCC is less documented than in viral infections. A recent meta-analysis showed that IL-10 levels in HCC patients increased compared to cirrhotic patients and healthy controls but not compared to viral hepatitis patients [53]. Individuals with resectable HCC and IL-10 levels > 12 pg/mL display worse postoperative outcomes [54]. A study showed that in unresectable HCC, the serum levels of IL-10 acted as a negative prognostic factor [19].

Tumor necrosis factor (TNF) (Table 1) is a cytokine produced by proteolytic cleavage from a transmembrane protein precursor (mTNF) into a soluble TNF (sTNF). sTNF binds TNF receptors (TNFR1) (constitutively expressed in most tissues) and TNFR2 (expressed only in hematopoietic and endothelial cells), while mTNF only the type 2 receptor. TNF induces numerous biological responses in the liver, such as apoptosis, necrosis hepatocytes, hepatic inflammation, and regeneration, as well as the progression of HCC [23]. Studies on mouse models provided the role of TNF in the immunopathogenesis of HCC by focusing attention on the transcription factor NF-kB (nuclear factor kappa–light–chain–enhancer of activated B cells) involved in the regulation of the pathway activated by the binding of TNF to the TNFR receptor [23]. In fact, a deficiency of the TNFR-dependent anti-apoptotic NF-kB signaling pathway seems to be essential for the induction of compensatory proliferation of alive hepatocytes in response to hepatocyte death, which results in the development of HCC [55]. Mice lacking the kB regulatory subunit of the kinase-function protein complex (IKK), particularly in hepatocytes, spontaneously develop a chronic liver disease that evolves into HCC [56]. Furthermore, specific deletion of hepatocyte IKKβ protein exacerbated chemically induced liver cancer in mice, possibly worsening carcinogen-induced hepatocyte death and induction of compensatory hepatocyte proliferation [57]. The role of TNFR2 receptors has not been widely analyzed; therefore, preclinical studies in chronic liver injury models are widely desired. The few studies on TNFR2 suggest that they may facilitate TNFR1-induced liver cell death. New-generation drugs against TNFR2 could be relevant for suppressing regulatory T-cell activity and, consequently, improving the efficacy of cancer immunotherapy [58]. A recent study has shown the correlation among polymorphisms of TNF-α and IL-10 genes as an increased risk of developing HCC in patients with chronic HCV infection, suggesting that gene variants are associated with more severe inflammation of the liver [59]. IL-10 is a cytokine with strong anti-inflammatory properties, which plays an important role in limiting the host’s immune response, thereby reducing damage and keeping the tissue in normal balance [60].

A study on HCV patients who have completed antiviral treatment with DAAs has shown the correlation between the genotyping of IL-10 (polymorphism IL-10 rs1800871) and the incidence of complications, such as HCC. This explains that the IL-10 genotype can help select the safest and most accurate drug regimen also based on the follow-up of the resistance of the genotype [59]. 

Jing et al. found that TNF-α overexpression promotes HCC through the activation of hepatic progenitor cells, while TNF-α deficiency inhibits the activation and proliferation of these cells, reducing tumor incidence. This confirmed that TNF-α plays a significant role in liver damage and prognosis [61]. 

Expression of IL-6 and TNF-α during chronic liver injury activates the transduction pathway downstream of the transcription factor STAT3, which drives neoplastic transformation in the hepatic microenvironment [62]. IL-6 mediates its pro-proliferative effects through activation and direct interaction with the p65 subunit of NF-kB, activation of which is associated with a frequent and early event in liver fibrosis and HCC, regardless of etiology [63,64].

### 2.3. Liver Tumor Inducers

IL-16 is a pleiotropic cytokine whose activity influences both the chemical attraction and the modulation of the activation of T lymphocytes (Table 1) [20]. It has been identified as an important over-expressed cytokine in human liver tissue of HCC in both non-tumor and tumor regions compared to benign tumors and non-cancerous liver levels. Furthermore, IL-16 production can activate the ERK (extracellular signal-regulated kinase)/cyclin D1 signaling pathway, leading to tumor growth [65]. 

Different research groups evaluate osteopontin as an early marker of HCC. Produced by Kupffer cells, stellate cells, and hepatocytes, this cytokine is highly expressed at sites of inflammation and tissue remodeling [66]. Osteopontin mediates a wide range of biological functions in the immune and vascular systems and has been extensively studied in numerous cancers [24]. An increase in serum osteopontin levels was found in individuals with HCC compared to liver cirrhosis alone or chronic liver disease. The specific diagnostic efficacy of osteopontin in detecting early-stage HCC by differentiating them from non-HCC patients varies considerably among studies. Two studies report that osteopontin levels within two years of diagnosis have a reasonable predictive value of HCC with an AUC (area under the curve) of 0.82 [67,68]. 

In a recent study, Askoura et al. described the role of interleukins IL-33, IL-17, and IL-25 in patients with HCV, the progression of the disease from chronicity to HCC, as well as the importance of using them as biomarkers of disease progression (Table 1). They measured serum levels of interleukins in HCV-related chronic hepatitis patients, HCC, and healthy controls. Their amounts were correlated with the degree of liver fibrosis and viral load. In contrast to serum levels of IL-25, which increased only in HCC patients, serum levels of IL-33 and IL-17 significantly increased in HCV and HCC patients. Furthermore, increasing serum levels of IL-33 seem to parallel the progression of liver fibrosis and viral load. The results indicate a significant role of IL-33 in liver inflammation and fibrosis progression in HCV infection, while IL-17 and IL-25 were featured as biomarkers for developing HCC [21].

After HCV eradication, patients undergo follow-up for the risk of developing HCC. Growth differentiation factor 15 (GDF15) is a cytokine, induced by mitochondrial dysfunction or oxidative stress (Table 1). In one study, serum levels of GDF15 were measured from patients with chronic HCV infection without a history of HCC who had achieved a sustained virological response with DAAs. Serum levels of GDF15 were higher in patients with HCC onset after treatment with DAAs than in untreated patients. Furthermore, the score obtained using an algorithm composed of the GDF15, AFP (alpha-fetoprotein), and the FIB-4 index stratifies the risk of developing HCC de novo after the elimination of HCV [22].

## 3. Detection and Measurement of Cytokines

The demand for increased testing, particularly for early events of hepatocellular carcinogenesis, for its recurrence or detection of minimal residual disease in those asymptomatic patients requiring alternative approaches. Different methods for measurement of cytokines are currently available, including immunoassays for the detection of single molecules (ELISA, western blot), multiplex assays (chemiluminescent, bead-based (Luminex), and planar antibody arrays), and mass spectrometry [69].

### 3.1. Enzyme Immuno Assays: EIA

As well as for diagnostics and clinical research, immunoassays represent the most often employed tests for detecting cytokines in biological fluids or cell culture media. The ELISA tests developed in 1971 are the most employed for their high specificity and sensitivity given by pairs of optimized antibodies and protein concentrations equal to 1–100 pg/mL (~10 times lower than the concentration of the most abundant plasma proteins), respectively [70]. 

The non-competitive ELISA, which uses a capture monoclonal antibody (primary), a biotinylated-detection monoclonal antibody (secondary), and a substrate complex of the streptavidin enzyme, is among the most widely used tests [71,72] and, although not as sensitive as biological tests, are more specific and have a quick and easy execution. Competitive EIA, unlike the non-competitive assay, is based on employing polyclonal capture antibodies and biotin-labeled ligands that compete for binding sites of the antibody with the sample ligand. Compared to the non-competitive assay, EIA is more sensitive, with high discriminating power in detecting free and protein-bound cytokines or soluble cytokine receptors [70,71,72].

### 3.2. Western Blot

Unlike ELISA, which determines the quantification of proteins in solution, Western Blotting is suitable for qualitative and semi-quantitative detection of cytokines by denaturing and separating them on the polyacrylamide gel, transferring to a nitrocellulose or polyvinylidene membrane difluoride, and, finally, quantifying using specific antibodies [73]. Although not as sensitive as ELISA, it adds data to the molecular weight of proteins and, therefore, can be used to determine splice variants or degradation of cytokine molecules. In addition, it may distinguish inactive precursors of cytokines from active forms characterizing the neoplastic environment [74]. It also allows the study of the phosphorylation sites of receptor proteins, e.g., the effects of VEGF-A and its inhibitor (bevacizumab) on cell proliferation, migration, invasion, and production of other cytokines in melanoma cell lines [75].

### 3.3. Electrochemiluminescence Immunoassays

Electrochemiluminescence (ECL), whose principle converts electrical energy into light emission, is based on the multi-array technology replaced by the ruthenium complex of the nineties [76]. ECL technology allows the simultaneous quantification of one to ten analytes on 96 or 384-well plates. Anti-cytokine antibodies are adherent to the surface of carbon electrodes, located on each well of the plate, and after the addition of the sample, the detection of cytokines occurs thanks to antibodies conjugated to an electro-chemiluminescent label (sulfo-tag). The passage of current through the carbon electrodes excites the sulfo-tags, and the light intensity will be directly proportional to the analytes in the sample. The ECL technique used both in clinical practice and scientific research, is based on the emission of a specific signal with an almost equal sensitivity compared to cytometry [77].

### 3.4. Luminex

The evolution of the ELISA is represented by multiplex assays that allow measuring multiple cytokines (for better disease monitoring) in the same reaction well, saving significant quantities of sample, time, and costs. The technique is based on antibody arrays using microspheres, such as Luminex assays, or planar arrays (antibody arrays and antibody microarrays) [78,79].

Both methods have high reproducibility, but recent studies have shown that the mean kit quality control coefficient of variation (CV) ranges from 1.9 to 18.2% for Luminex and 2.4 to 13.9% for Planar Antibody Array and that the latter has a lower limit of quantification (LLoQ) than Luminex [80].

Such assays can measure up to 100 analytes in parallel, but the possibility of cross-reactivity with antibodies has reduced the dosage to 30 analytes. Luminex assays provide high throughput, precision, and sensitivity, reaching pg/mL concentrations and reproducibility achieved with 25–50 µL of the sample [81].

### 3.5. Planar Antibody Array

The sandwich-based Planar antibody array methodology uses pairs of antibodies tested to eliminate cross-reactivity to any other antigen / antibody in the array. Therefore, high sensitivity, specificity, and productivity of the technique allows to detect between 10 and 80 analytes up to the quantification of 1000 secreted proteins including cytokines and chemokines [78].

Antibodies immobilized on nitrocellulose membranes or on the glass slides allow easy semi-quantitative and quantitative measurements with fluorescent detection. 

The methodology finds application above helpful in the search for possible diagnostic and prognostic biomarkers based on the development of the tumorigenesis process and tumor progression [82].

### 3.6. Mass Spectrometry (MS)

This proteomics technique involves several steps, including the isolation of proteins, digestion by proteases into smaller peptides, concentration and removal of salts, separation by high-performance liquid chromatography (HPLC), and ionization based on the mass-to-charge ratio (m/z) of peptides [83].

The high specificity of the methodology in the identification and quantification of peptides/proteins of MS is contrasted by the low sensitivity due to the pretreatment of the sample by the cytokines with the lowest molecular weight that negatively influence the laborious and difficult ionization process, such as to provide a low yield of peptides and, consequently, poor detection of these cytokines. Therefore, MS offers a higher multiplexing capacity but lower sensitivity than immunoassays [83]. 

### 3.7. Challenging Frontiers of Analytic Methods

Thanks to advanced technologies, ultra-sensitive methods that improve the sensitivity of the analytic range down to the order of femtoliters and reduce the effects of the sample matrix and minimization of sample volumes are currently available. Starting from traditional colorimetric, chemiluminescent, and fluorescent detection, laboratory diagnostics are now moving towards electrochemical, optical, mechanical, or surface plasmon resonance measurement biosensing [84,85,86].

Extracellular vesicles are plentifully released into the systemic circulation, where they harbor molecules that provide biochemical information about their cells of origin [87]. 

Extracellular vesicles represent a challenging source of circulating analytes for cancer liquid biopsy. A promising diagnostic alternative in precision medicine is noninvasive and can be done more frequently than a tissue biopsy. 

## 4. Conclusive Remarks

HCC is a major healthcare burden with a high prevalence and poor prognosis. The identification of molecules as biomarkers for early cancer detection and therapeutic targets for cancer treatment is an important issue in precision medicine. 

In clinical practice, screening high-risk patients with ultrasound and/or alpha-fetoprotein dosage is widespread. Now, the reduction in mortality is related to viral infection control measures.

In this scenario, it is crucial for clinicians to provide benefits for HCC therapies. Targeting the hallmarks of cancer represents one of the approaches to anchoring this problem. For HCC, hallmarks include maintenance of proliferative signaling, avoidance of growth suppressors, escape immune destruction, replicative immortality, promotion of inflammation, activation of invasions and metastases, inducing angiogenesis, mediating genome instability and mutation, resisting cell death, and deregulating cellular energy [88]. This means that more hallmarks, pathways, and cytokines are involved.

Currently, the molecular mechanisms underlying HCC remain partly enigmatic. Studies based on the so-called “omics” sciences (e.g., transcriptomics, proteomics, metabolomics) facilitate the learning of global changes in molecules in a given disease in a high-throughput way and, therefore, are suitable for understanding the complex changes that lead to the onset and evolution of HCC.

In the premalignant environment, inflammatory cells release a multitude of cytokines, chemokines, growth factors, prostaglandins, and proangiogenic factors, making the liver environment a favorable zone for hepatocyte transformation from an accumulation of genetic mutations. Survival of transformed hepatocytes is possible through the activation of anti-apoptotic pathways and suppression of immune surveillance [89], as shown in Figure 2A,B. VEGF, stimulated by HIF-1α induced in the hypoxic environment, plays a central role [6].

The risk of developing HCC increases with the severity of liver inflammation and fibrosis. Chronic inflammation is sustained by a range of inflammatory mediators also identified as a cause of carcinogenesis [90]. A complex interaction of several pro-inflammatory cytokines (IL-6, TNF-α) and anti-inflammatory cytokines (TGF-α and β), different transcription factors (NF-kB, STAT-3), and their signaling pathways are involved in the development of HCC [68,91]. 

We recently found that angiogenic markers, with emphasis on Ang1/2, may contribute to developing quantitative tools for liver disease staging and therapy monitoring [9]. A comparison of cirrhotic patients with and without HCC suggests that HGF levels are potentially useful for monitoring the onset of HCC after a diagnosis of cirrhosis. Elevated Ang1 levels in HCC patients appear to have a protective role, as well as prognostic significance [9].

Characterization of the expression profile of tumor-associated inflammatory cytokines in HCC will require novel diagnostic and therapeutic strategies, such as a better understanding of cytokine regulatory mechanisms in the hepatic microenvironment.

Early diagnosis of HCC remains the goal of precision medicine. The laboratory participates in the follow-up of the patient at risk of a neoformation or recurrence, after any surgical resection, by measuring the cytokines that activate gene transcription of the cell to produce an adequate response to that stimulus. Therefore, the measurement of biomarkers can represent an accurate diagnostic tool for the oncologist to be used alongside imaging procedures.

## Data Availability

Data sharing is not applicable to this article as no datasets were generated or analyzed during the current study.

## Abbreviations

AFP (alpha-fetoprotein); Ang (angiopoietin); AUC (area under the curve); DAAs (direct antiviral drugs); ECL (electrochemiluminescence); EIA (enzyme immunoassay); ELISA (enzyme-linked immunosorbent assay); ERK (extracellular signal-regulated kinase); FAK (focal adhesion kinase); GDF15 (growth differentiation factor 15); HCC (hepatocellular carcinoma); HGF (hepatocyte growth factor); HIF-1α (hypoxia-inducible factor 1a); HPLC (high-performance liquid chromatography); IKK (kinase-function protein complex); LLoQ (lower limit of quantification); MELD (model for end stage liver disease); MS (mass spectrometry); NAFLD (non-alcoholic fatty liver disease); NF-kb (nuclear factor kappa-light-chain-enhancer of activated B cells); NASH (non-alcoholic steatohepatitis); PECAM-1 (platelet endothelial cell adhesion molecule-1); SMAD (small mother against decapentaplegic; STAT-3 (signal transducer and activator of transcription); TGF (tumor growth factor); Tie 1 and 2 (tyrosine kinase with Ig and EGF-omology domains-1 receptors); TNF (tumor necrosis factor); Vascular endothelial growth factor (VEGF).
